# Cochlea-inspired design of an acoustic rainbow sensor with a smoothly varying frequency response

**DOI:** 10.1038/s41598-020-67608-z

**Published:** 2020-07-01

**Authors:** Angelis Karlos, Stephen J. Elliott

**Affiliations:** Institute of Sound and Vibration Research, University of Southampton, Highfield Campus, Southampton, SO17 1BJ UK

**Keywords:** Engineering, Physics

## Abstract

A number of physical arrangements for acoustic rainbow sensors have been suggested, where the aim is to separate different frequency components into different physical locations along the sensor. Although such spatial discrimination has been achieved with several designs of sensor, the resulting frequency responses at a given position along the sensor are generally not smoothly varying. In contrast, the cochlea provides an interesting natural example of a rainbow sensor, which has an exponential frequency distribution and whose response does vary smoothly with frequency. The design of a rainbow sensor is presented that has a number of discrete resonators and an exponential frequency distribution. We discuss the conditions for a smoothly varying frequency response in such a sensor, as part of a broader design strategy. It is shown that the damping within the resonators determines the trade-off between the frequency resolution and the number of elements required to achieve a smooth response. The connection is explained between this design and that of an effective acoustic absorber. The finite number of hair cells means that the cochlea itself can be thought of as being composed of discrete units and the conditions derived above are compared with those that are observed in the cochlea.

## Introduction

Acoustic rainbow sensors consist of arrays of units of sub-wavelength dimensions, whose properties vary with position, which, through strong wave dispersion, can enhance and spatially separate different frequency components of incident acoustic waves^1^. The sub-wavelength dimensions of the unit elements of acoustic rainbow sensors and their strongly dispersive behaviour, which is not encountered in natural materials, classify them in the realm of acoustic metamaterials^2^.

For an acoustic rainbow sensor to be effective as a spectrum analyser, the frequency response at a given position needs to have the form of a band-pass filter with a peak at a certain frequency and without additional peaks within the working frequency range. Although several designs of acoustic rainbow sensor have previously been proposed, they generally do not have responses that vary smoothly with frequency, and therefore do not have an unambiguous peak in their frequency response. One-dimensional rainbow sensors consisting of narrow grooves of varying depth perforated in a rigid substrate have previously been presented^1,3^, using the quarter-wave resonance within the groove. In those designs, it was demonstrated both by simulations and experimentally that incident waves slow down and get amplified as they propagate along the system, until they reach a position, which is different for different frequency components, where they are trapped, beyond which they are greatly attenuated. However, the experimentally observed pressure envelope did not vary smoothly either with position or with frequency. A more compact system using coiled quarter-wave resonators was presented in^4^, but the frequency response in the resonators was not discussed. A two-dimensional system, similar to that in^1^ but consisting of perforations of varying depth over a planar substrate, was presented in^5^. Viscous and thermal losses were included in the simulations, which predicted a smoother frequency response in terms of the acoustic pressure, although with some fluctuations with frequency.

A design consisting of a duct with an array of side branches of Helmholtz resonators with varying cavity volume was proposed by Zhao and Zhou^6^, where the coiling of the system was loosely based on the form of the cochlea. Simulations predicted the rainbow trapping effect, although again with significant fluctuations in the pressure modulus response, since no losses were accounted for. A similar array of Helmholtz resonators designed as an acoustic absorber was presented by Jimenez et al.^7^, where the dimensions of both the resonators and the main duct vary with position. The geometrical parameters of this system were numerically optimised in order to maximise the absorption over a given frequency band. Very high absorption was demonstrated experimentally and, as a side-effect, the enhancement of different frequency components at different resonators was predicted.

One motivation for designing a system with the response characteristics of the cochlea stems from the higher efficiency of such a wave-based spectral analysis, compared to the Fast Fourier Transform algorithm or a filter bank. Specifically, it was shown in^8^ that, for the discrimination of *N* frequencies, the required analysis time, power and hardware usage of the wave-based spectral analysis in the cochlea scales proportionally to *N* compared to proportionally to $N\log N$ for the Fast Fourier Transform or $N^2$ for a filter bank. Also, although in many current applications advanced digital systems currently appear to provide the most effective frequency analysers, it is still worthwhile to investigate alternative acoustic or mechanical^9^ technologies for future or niche applications. In the acoustic regime, a nonlinear acoustic rainbow sensor with side branches of quarter-wave resonators was recently presented^10^, which aims to mimic the response characteristics of both the passive cochlea, at high input levels, and the active cochlea, at low input levels. In this case, the passive system had a smoothly varying frequency response but the active system did not, which the authors attribute to reflected waves at low levels and postulate that these may also occur in the cochlea. It is thus important to investigate the characteristics of acoustic rainbow sensors that result in them having a smoothly varying frequency response.

This article presents a physically motivated design of an acoustic rainbow sensor consisting of a main duct of constant cross section with an array of Helmholtz-resonator side branches of varying dimensions. The design principles are based on the response characteristics of the cochlea as a rainbow sensor, where different frequency components are focused at different positions with an approximately exponential mapping of frequencies along its length^11^. In particular, the designed rainbow sensor aims to reproduce the smooth frequency response of the cochlea when measured at a given position, which has an unambiguous peak^12^. The main objective of the article is to explore the trade-offs inherent in this design in order to reproduce the smooth frequency response of the cochlea. It is shown that in order to ensure a smoothly varying frequency response, a minimum number of side branches per octave of resolved frequencies must be used. This minimum number depends on the number of cycles of phase shift at resonance and the quality factor of the Helmholtz resonators. The design principles are established using simple lumped-parameter models, but the response of an example sensor is then calculated using a more accurate distributed-parameter Transfer Matrix method. The trade-off between achieving a smooth response and minimising the number of elements is explored both theoretically and in simulations.

## Results

**Fig. 1:**
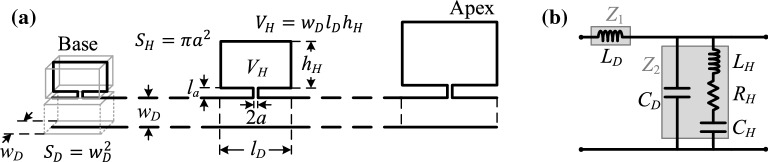
Schematic of a cochlea-inspired acoustic rainbow sensor. (**a**) Longitudinal cross section along the mid-width plane of a system consisting of a square-cross-section duct of side $w_D$, with side branches consisting of Helmholtz resonators of varying dimensions. The neck radius, *a*, and length, $l_a$, are shown, along with the cavity volume, $V_H$. Each element of the system consists of a duct segment of length $l_D$ and a Helmholtz resonator. The length and width of each cavity are the same as those of the corresponding duct segment, and the height of each cavity is denoted by $h_H$. Formulas for the cross-sectional area of the duct, $S_D$, and of the neck, $S_H$, are shown, as well as for the volume of the cavity. The left and right ends of the system correspond to the base and the apex of the cochlea, respectively. (**b**) Circuit representation of an element of the system. $Z_1$ is the series impedance, consisting of the inertance of the duct, $L_D$, and $Z_2$ is the shunt impedance, consisting of the duct compliance, $C_D$, in parallel with an RLC branch representing the Helmholtz resonator, with resistance $R_H$, inertance $L_H$ and compliance $C_H$.

The properties of the passive cochlea can be reasonably well reproduced if one-dimensional fluid coupling is assumed, together with single-degree-of-freedom dynamics of the cochlear partition, with an exponential distribution of natural frequencies^13^. An entirely acoustic system can also be designed to reproduce the behaviour of the cochlea, consisting of discrete elements, provided that a sufficiently large number of elements is used. Such a system, consisting of a main duct of constant cross section with Helmholtz-resonator side branches of varying geometrical properties, is shown in Fig. 1a, where the input position is called ‘the base’ and the terminating position ‘the apex’, by analogy with the cochlea. For the design of such a system, lumped-parameter acoustic modelling is used. A discretised Transmission Line approximation for an array of identical Helmholtz resonators is first used to obtain the wavenumber and from that, an approximate formula for the minimum wavelength in the waveguide. Formulas derived from this analysis are then used in the design of a system whose element dimensions vary with position, assuming that these vary relatively smoothly.

### Transmission Line analysis of an array of identical Helmholtz resonators

A system consisting of a main duct with an array of identical Helmholtz-resonator side branches is first considered, where each element of the system comprises a segment of the main duct of constant length $l_D$ and a side branch of a Helmholtz resonator. Such a system produces a ‘slow wave’, that is, a wave whose propagation speed is smaller than that of an acoustic plane wave in a uniform tube, up to a frequency where the propagation speed becomes very small^14^. Above this frequency, there is a stop band, where the wavenumber is mainly imaginary. Beyond this, there is another pass band, in which waves propagate with a speed that approaches the speed of plane-wave propagation as frequency increases. These propagation characteristics will also be observed in the designed system below, although the aim of the current analysis is to provide a simple model of the wave propagation that can be used for design purposes.

Assuming that the dimensions of the components of an element are much smaller than the wavelength, an equivalent circuit with lumped parameters can be used^15^ and the system can be analysed using Transmission Line theory, as presented for example in^16^. This assumption also ensures that only the fundamental mode within the duct can propagate in the considered frequency range. An equivalent circuit representation of an element of the system is shown in Fig. 1b, in which all the impedances are acoustic, that is, ratios of acoustic pressure to volume velocity. The element lumped parameters in Fig. 1b correspond to those of a finite duct length, whereas in classic Transmission Line theory, the lumped parameters of each infinitesimal segment of the waveguide represent inertances, compliances and resistances per unit length of the Transmission Line. Therefore, the ratio of each lumped parameter to the element length, $l_D$, corresponds to the parameter per unit length used in Transmission Line theory.

The series impedance of each element is only due to the inertance of the duct segment and is denoted by $Z_1$, whereas the shunt impedance, denoted by $Z_2$, consists of the parallel combination of the compliance of the air in one section of the duct and the impedance of the Helmholtz resonator. The resistance, inertance and compliance of the Helmholtz resonator, as derived in the Supplementary material, are given by

1 $$\begin{aligned} R_H = \frac{\alpha \rho c_0 l_H}{S_H}, L_H = \frac{\rho l_H}{S_H}\;\text {and}\;C_H = \frac{V_H}{\rho c_0^2}, \end{aligned}$$

where $l_H$ is the effective neck length, $S_H$ is the neck cross-sectional area, $V_H$ is the cavity volume, $\rho$ is the air density, $c_0$ is the speed of sound in air and $\alpha$ is the frequency-dependent loss coefficient. It should be noted that the effective neck length, $l_H$, is larger than the actual neck length, $l_a$, shown in Fig. 1a, since the former also includes end corrections, as explained below. The inertance and compliance of the duct are given by $L_D=\rho l_D/S_D$ and $C_D=V_D/\rho c_0^2$, where $V_D=S_Dl_D$ is the volume of the duct segment. Viscous and thermal losses are only considered in the neck of the Helmholtz resonator. It should be noted that the acoustic resistance, $R_H$, depends on frequency. The element impedances can be expressed in terms of the lumped parameters, assuming an ${\mathrm {e}}^{{\mathrm {i}}\omega t}$ time dependence, as $Z_1={\mathrm {i}}\omega L_D$ and $Z_2=Z_{C_D}Z_H/\left( Z_{C_D}+Z_H\right)$, where $Z_{C_D}=-{\mathrm {i}}/\omega C_D$ and $Z_H$ is given by

2 $$\begin{aligned} Z_H = R_H + {\mathrm {i}}\omega L_H + \frac{1}{\mathrm {i}\omega C_H}. \end{aligned}$$

The Helmholtz resonance angular frequency, when ${\mathrm {i}}\omega L_H$ is equal to $-{\mathrm {i}}/\omega C_H$, and the corresponding quality factor are given by^17,18^ $\omega _1=c_0\sqrt{S_H/l_H V_H}$ and $Q_1=\omega _1 L_H/R_H(\omega _1)$, where the acoustic resistance is evaluated at the resonance frequency.

The wavenumber of the Transmission Line is given by^16^

3 $$\begin{aligned} k = \frac{1}{{\mathrm {i}} l_D}\sqrt{\frac{Z_1}{Z_2}}, \end{aligned}$$

where ${\mathrm {i}}k$ corresponds to the propagation constant of the Transmission Line. The wavenumber in Eq. (3) corresponds to a wave travelling along the system as if it were a uniform Transmission Line. In other words, the duct with the array of identical Helmholtz resonators is transformed into a uniform acoustic waveguide, whose properties are smeared out so that they do not change with position. A cut-on angular frequency is also introduced, which forms the upper limit of the stop band, defined by $\omega _2=\omega _1\sqrt{1+H}$, where $H=C_H/C_D=V_H/V_D$, and a second quality factor is defined as $Q_2=Q_1\omega _2/\omega _1$. By substituting the relations for $Z_1$ and $Z_2$ given above into Eq. (3), also using the expressions for $\omega _1$, $Q_1$, $\omega _2$ and $Q_2$, the wavenumber takes the relatively simple form

4 $$\begin{aligned} k = k_0\left[ \frac{\omega _2^2 + {\mathrm {i}}\frac{\omega \omega _2}{Q_2} - \omega ^2}{\omega _1^2 + {\mathrm {i}}\frac{\omega \omega _1}{Q_1} - \omega ^2}\right] ^{1/2}, \end{aligned}$$

where $k_0=\omega /c_0$. At very low frequencies, that is, for $\omega \ll \omega _1$, the wavenumber tends to the value $k_b=k_0\omega _2/\omega _1$, where the subscript *b* has been used in correspondence with the limiting behaviour at the basal end, as will be seen below, whereas at very high frequencies, for $\omega \gg \omega _2$, the wavenumber tends to that in free space, $k_0$. For intermediate frequencies where $\omega _1\ll \omega \ll \omega _2$, the wavenumber takes the form $k\approx -{\mathrm {i}}/d$, where $d=c_0/\omega _2$ is the exponential decay length, so that a stop band is formed between $\omega _1$ and $\omega _2$, where waves do not propagate but are attenuated.

The wavelength of the wave propagating in the Transmission Line is given by $\lambda =2\pi /{\mathrm {Re}}\{k\}$, whereas the wavelength in free space is given by $\lambda _0=c_0/f=2\pi /k_0$. Assuming that the band gap is large, that is, $\omega _2\gg \omega _1$, the wavenumber at the resonance frequency of the Helmholtz resonators takes the approximate form $k(\omega _1)\approx \sqrt{Q_1/2}(1-{\mathrm {i}})/d$. The wavelength reaches a minimum at a frequency which is close to the resonance frequency $\omega _1$, as will be seen below, and so is approximately given by

5 $$\begin{aligned} \lambda _{min} \approx \frac{2\pi }{{\mathrm {Re}}\{k(\omega _1)\}} \approx 2 \pi d\sqrt{\frac{2}{Q_1}}. \end{aligned}$$

For air in room temperature and a neck radius much larger than the viscous boundary layer thickness, the loss coefficient is approximately given by $\alpha \approx 3\cdot 10^{-5}\sqrt{f/{\mathrm {Hz}}}/a$, where *f* is the frequency in Hz and *a* is the neck radius, so that if *a* is in metres, then $\alpha$ is in Np/m, as derived in the Supplementary material. Using this approximate formula for the loss coefficient and the expressions for $R_H$, $L_H$ and $Q_1$, the quality factor takes the simple approximate form

6 $$\begin{aligned} Q_1 \approx 600 (a/{\mathrm {m}})\sqrt{f_1/{\mathrm {Hz}}}, \end{aligned}$$

where $f_1=\omega _1/2\pi$ is the resonance frequency in Hz, and *a* is the neck radius in meters.

The effective neck length, $l_H$, is equal to the sum of its physical length, $l_a$, and end corrections at both ends. For the inertance, these end corrections account for the mass of air outside the neck that gets accelerated by the air within it^19^, whereas for the resistance, the end corrections account for the additional loss as the air flows around the two ends of the neck^18^. For this design study, it is assumed that the end correction for the resistance is equal to that for the inertance. The end correction for a flanged end is approximately^18^ 0.85*a*, although more complicated formulas have also been proposed for a baffle of finite size^14^, presented in the Supplementary material, which are used in the simulations.

### Design requirements and trade-offs for an acoustic rainbow sensor with varying Helmholtz resonators

An acoustic system with Helmholtz-resonator side branches of varying dimensions can be designed by analogy with the varying stiffness and mass of the basilar membrane along the cochlea. The varying dimensions of the cavities of the Helmholtz resonators correspond to the variation of the stiffness and the varying lengths of the necks to the variation of the mass. The longitudinal inertia of the liquid in the cochlear chambers, which provides the longitudinal coupling, is accounted for by the inertia of the air in the main duct. If the dimensions are assumed to vary relatively smoothly between consecutive elements, the wavenumber of the system will be approximately given by Eq. (4), where now the wavenumber will also depend on position, since the resonance frequency, $\omega _1$, will now vary for the different Helmholtz resonators, and accordingly $\omega _2$, $Q_1$ and $Q_2$ will also vary with position.

For a discrete system to accurately approximate a continuous waveguide, a minimum number of elements per wavelength along the direction of propagation has to be used, with a minimum of six elements per wavelength often applied, as for example in Finite Element Analysis^20^. In the system of a duct with an array of Helmholtz resonators, the condition of at least six elements per wavelength has to be satisfied for the minimum occurring wavelength. The decay length, defined as $d=c_0/\omega _2$ above, can also be written as $d=l_D\sqrt{\mu H/(1+H)}$, where $H=V_H/V_D$, and $\mu =l_HS_D/l_DS_H$ is the ratio of the inertance of the Helmholtz resonator to that of the duct segment. The minimum wavelength given by Eq. (5) then takes the form $\lambda _{min}\approx 2\pi l_D\sqrt{2\mu H/Q_1(1+H)}$.

In the cochlea, the tonotopic mapping of the local natural frequency of vibration of the basilar membrane with position can be approximated by^12^ $f_n(x)=f_B {\mathrm {e}}^{-x/l}$, where $f_B$ is the natural frequency at the base and *l* is the lengthscale of the exponential decrease in frequency. If an equivalent discrete system with equally spaced elements is considered, then the natural frequency of the *n*-th element is given by $f_1(n)=f_B{\mathrm {e}}^{-(n-1)l_D/l}$. A constant element length, $l_D$, is assumed here to estimate the distribution of elements per octave. The number of elements per octave is calculated as the difference in the order of two elements whose natural frequencies are an octave apart, that is, $N_{oct}=n_2-n_1$, where $f_1(n_1)=2f_1(n_2)$. Using the discrete natural frequency variation above, the number of elements per octave is calculated as $N_{oct}\approx 0.7l/l_D$.

A more meaningful expression for the appropriate number of elements per octave can be found by combining two requirements. First, the minimum wavelength as expressed in Eq. (5) can be conveniently set to be equal to $\lambda _{min}=2\pi l_D\gtrsim 6l_D$ to satisfy the smoothness requirement, of at least six elements per minimum wavelength, so that the element length can be written as $l_D=d\sqrt{2/Q_1}$. Also setting $\lambda _{min}=2\pi l_D$ in the relation $\lambda _{min}\approx 2\pi l_D\sqrt{2\mu H/Q_1(1+H)}$ derived above requires that

7 $$\begin{aligned} \mu =\frac{1+H}{2H}Q_1. \end{aligned}$$

For a given $Q_1$, Eq. (7) leads to a restrictive condition for the dimensions of the Helmholtz resonators in relation to the dimensions of the duct segment involved in the definition of $\mu$, a condition which is used in the design procedure. The second requirement is that the number of cycles of phase shift should be approximately given by the non-dimensional phase-shift parameter introduced for the cochlea in^21^, and defined as $N_c=l/4d$, which also defines the form of the frequency response^22^. Combining this expression with the result $N_{oct}\approx 0.7l/l_D$ for the number of elements per octave, also using the expression $l_D=d\sqrt{2/Q_1}$, gives a relation for the number of elements per octave, for a given $N_c$ and $Q_1$, as

8 $$\begin{aligned} N_{oct} \approx 2 N_c \sqrt{Q_1}. \end{aligned}$$

Therefore, the estimation of an appropriate number of elements per octave stems from the smoothness requirement of sufficient elements per wavelength and from the required form of the frequency response.

It is clear from Eq. (8) that, for a given $N_c$, the choice of the damping in the resonators thus involves a trade-off in the design between good frequency selectivity, provided by a narrow band-pass frequency response in the case of high $Q_1$, and the number of elements required for a given frequency range to give a smooth response. The attenuation per octave in the stop band can also be calculated as

9 $$\begin{aligned} {\mathrm {Att_{oct}}}={\mathrm {e}}^{-N_{oct}l_D/d}\approx {\mathrm {e}}^{-2.8N_c}, \end{aligned}$$

where Eq. (8) and the relation $l_D=d\sqrt{2/Q_1}$ have been used. Therefore, for a given $N_c$, the required total attenuation within the stop band will define the width of the band gap. This will then impose a minimum value for *H* in the design, which is the ratio of the volume of the cavity to that of the duct, since $\omega _2/\omega _1=\sqrt{1+H}$.

### A design example

The phase-shift parameter is chosen to be $N_c=2.5$, which is a good approximation for the phase shift in the human cochlea^12^. For this value of $N_c$, Eq. (9) gives an attenuation in dB of approximately 60 dB/octave in the stop band. If it is assumed that an attenuation of 30 dB within the stop band is sufficient, then a band gap of at least half an octave should be used, that is, $f_2/f_1\ge \sqrt{2}$, where $f_2=\omega _2/2\pi$. This means that $H\ge 1$, which requires that the volume of a given Helmholtz resonator has to be larger than or equal to the volume of the corresponding duct segment.

The example system considered here is designed to work in the standard telecommunications band of 300 Hz to 3.4 kHz, which is about 3.5 octaves wide. Therefore, the basal and apical natural frequencies are set to be $f_B = 3.4$ kHz and $f_A = 300$ Hz. Assuming a neck radius of $a = 0.5$ mm for all the elements, to ensure a reasonable fidelity of manufacturing using conventional 3D printing^7^, the quality factor for the first and the last element are calculated from Eq. (6) to be $Q_{1,B} = 17.5$ and $Q_{1,A} = 5.2$. The number of elements per octave, calculated by Eq. (8) with the respective values for $Q_1$ for the basal and apical elements, takes approximately the values 21 and 11. On average, it can be assumed that around 15 elements per octave are required, so that for the 3.5 octaves of the considered bandwidth, around 50 elements are required in total.

**Fig. 2:**
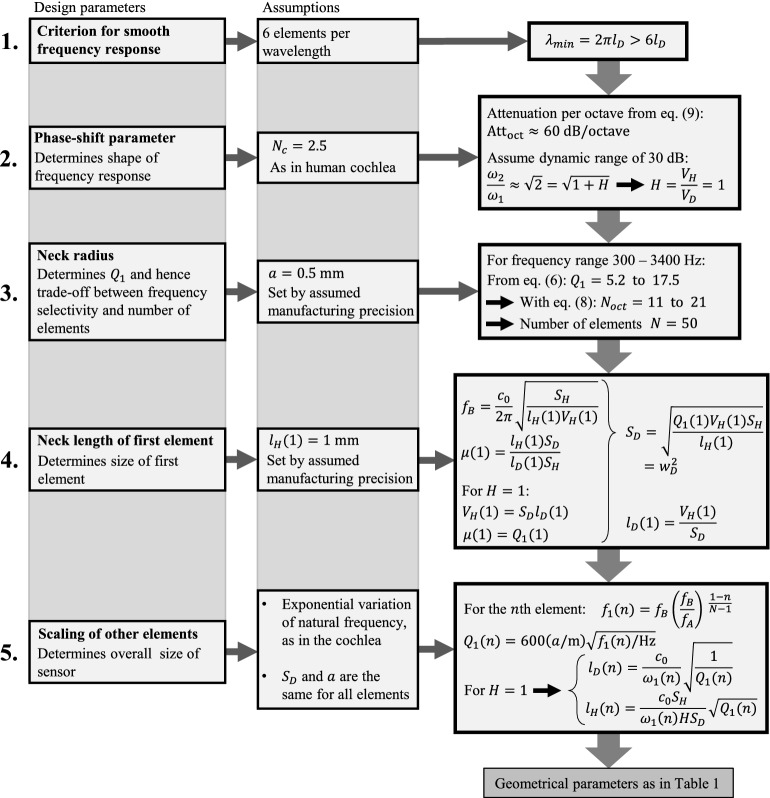
Flow chart of the design procedure of the acoustic rainbow sensor.

An effective neck length of $l_H=1$ mm is assumed for the first element, which, using the relation for $\omega _1$ and the relations $V_H=Hl_DS_D$ and $\mu =l_HS_D/l_DS_H$, along with Eq. (7) with the minimum required value $H=1$, defines the values for the length, $l_D$, of the first element and for the cross-sectional area of the main duct, $S_D$. The side of the square-cross-section duct, which is constant throughout the system, is then calculated by $w_D=\sqrt{S_D}$. An exponential variation of the resonance frequencies of the Helmholtz resonators is defined, by analogy with the cochlea, as $f_1(n)=f_B(f_B/f_A)^{(1-n)/(N-1)}$, which also defines the respective quality factors according to Eq. (6). Using the same relations as for the first element along with the defined resonance frequencies and quality factors, the element length, $l_D(n)$, and the effective neck length, $l_H(n)$, of the other elements can be calculated by $l_D(n)=\sqrt{2/(1+H)Q_1}c_0/\omega _1$ and $l_H(n)=\sqrt{(1+H)Q_1/2}c_0S_H/\omega _1HS_D$. Table 1 indicatively shows the parameters for the first, 25th and 50th element for this design example. The design procedure is illustrated as a flow chart in Fig. 2.

**Table 1:** Design parameter values of the acoustic rainbow sensor. Main parameter values of the first, 25th and 50th element of a system with an array of 50 Helmholtz resonators of varying dimensions. The neck radius is $a=0.5$ mm and the duct width is $w_D=7.3$ mm for all the elements.

*n*	$f_1$ (Hz)	$Q_1$	$l_D$ (mm)	$h_H$ (mm)	$l_a$ (mm)	$l_H$ (mm)
1	3,400	17.5	3.8	7.3	0.31	1
25	1,035	9.7	16.9	7.3	1.7	2.4
50	300	5.2	79.8	7.3	5.42	6.2

### Simulations

A set of results for the design example with 50 elements is presented here, analysed using a distributed-element Transfer Matrix method. This is a more accurate model than the lumped-parameter approach used for the design, since it accounts for wave propagation within the elements, and also includes losses in the main duct and in the cavities, and is used to provide an independent check on the assumptions made in the design process. The input of the system is a harmonic volume velocity of constant amplitude $2\cdot 10^{-7}$ m$^3\,$s$^{-1}$ at the left side of the first element. A matched load is assumed as the termination of the main duct, although results presented in the Supplementary material show that this is not essential.

#### Wavenumber and acoustic pressure

**Fig. 3:**
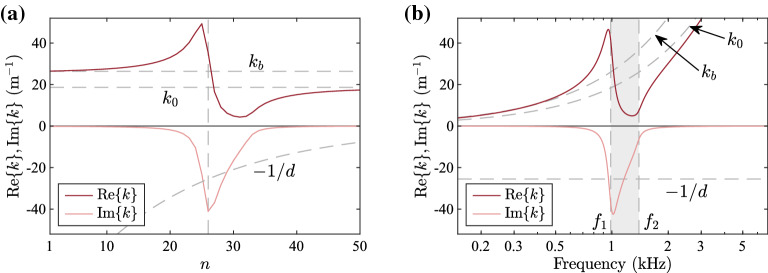
Wavenumber plots for the design example of an acoustic rainbow sensor. (**a**) Real and imaginary parts of the wavenumber plotted against the number of element, *n*, for a frequency of 1 kHz. (**b**) Real and imaginary parts of the wavenumber plotted against frequency for the 26th element. In (**a**), the vertical line corresponds to the the 26th element, whose resonance frequency, 985 Hz, is the closest to the input frequency. In (**b**), $f_1$ is the resonance frequency of the element used and $f_2$ is the corresponding cut-on frequency. The frequencies $f_1$ and $f_2$ form the limits of the stop band, which is shaded in grey.

The real and imaginary parts of the wavenumber calculated using Eq. (4) are plotted against position, for a frequency of 1 kHz, and against frequency, for the 26th element, in Fig. 3a, b, respectively. At basal positions and lower frequencies, the wave propagates with a purely real wavenumber whose value tends to $k_b=k_0\omega _2/\omega _1$, which is larger than $k_0$ and therefore corresponds to smaller propagation velocity, plots for which are included in the Supplementary material. At apical positions and higher frequencies, the wavenumber is again purely real but tends to the free-space wavenumber, $k_0$. Between these two pass bands defined by the resonance frequency, $f_1$, of a given element and the corresponding cut-on frequency, $f_2$, a stop band can be seen where the imaginary part of the wavenumber has large negative values so that the waves are attenuated. A spatial region of evanescence also occurs beyond an element which is basal to the element of resonance for a given frequency, as can be seen in Fig. 3a. In the stop band, the imaginary part of the wavenumber is seen to be of the order of $-\,1/d$, as presented above. Even though the stop band is not wide enough for any extended frequency range to satisfy the condition $\omega _1 \ll \omega \ll \omega _2$, and therefore the imaginary part of the wavenumber only has the value $-\,1/d$ close to the middle of the stop band, this still provides a reasonable approximation for the average value along the band.

**Fig. 4:**
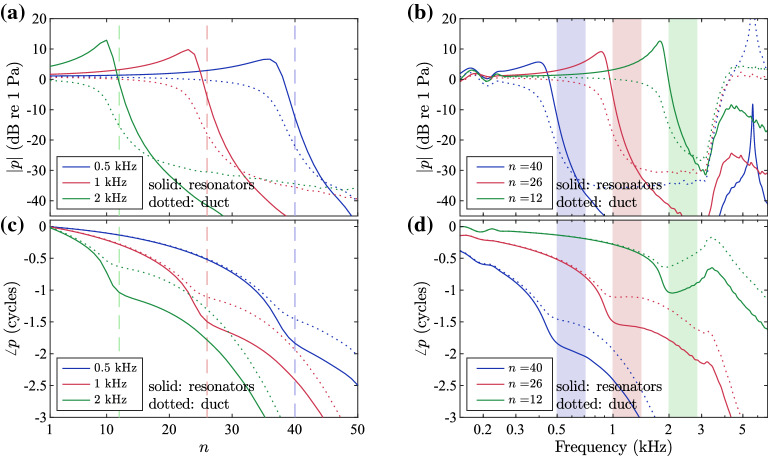
Pressure plots for the design example of an acoustic rainbow sensor. (**a**) Modulus of the pressure plotted against the number of element, *n*, for three different frequencies. (**b**) Modulus of the pressure plotted against frequency for three different elements. (**c**) Phase of the pressure plotted against *n* for three different frequencies. (**d**) Phase of the pressure plotted against frequency for three different elements. In (**a**) and (**c**), the coloured dashed vertical lines correspond to the elements whose resonance frequency is the closest to the respective input frequency; specifically, elements 12, 26 and 40 have resonance frequencies 1.97 kHz, 985 Hz and 492 Hz, respectively. In (**b**) and (**d**), the shaded areas correspond to the stop bands of the plot lines of similar colour. In all graphs, the solid lines correspond to the pressure in the resonators and the dotted lines to the pressure in the main duct.

Figure 4a shows the predicted variation of the modulus of the acoustic pressure along the waveguide calculated using the Transfer Matrix method, both at the top of the cavities in the resonators and in the main duct, plotted against the element number, *n*, for three different frequencies. It can be seen that the pressure in the resonators builds up smoothly along the waveguide, reaching a peak at a different element for different frequencies, after which it decreases rapidly, with lower frequencies peaking at apical positions and higher frequencies peaking at basal positions. This pattern for the pressure in the resonators closely resembles the coupled response of the cochlea in terms of the transverse velocity of the basilar membrane^12^. The pressure in the duct, plotted in dotted lines, decreases slowly up to the position where the pressure in the resonators peaks, beyond which it also rapidly decays, which is similar to the variation of the pressure difference across the basilar membrane, as can be seen for example in^23^. These results justify the correspondence of the side branches with the basilar membrane and of the main duct with the fluid-filled cochlear chambers.

The modulus of the pressure is also plotted against frequency in Fig. 4b for three different elements. It can be seen that within the frequency range defined by the basal and apical resonance frequencies, that is, between 0.3 kHz and 3.4 kHz, the pressure in the resonator of a specific element smoothly increases and peaks at a specific frequency, with basal elements peaking at higher frequencies and apical elements peaking at lower frequencies, similarly to the cochlea^12^. The peaks of the resonator pressures occur at lower frequencies compared to the corresponding resonance frequencies, denoted by the grey vertical dashed lines, due to the presence of damping. At lower frequencies, damping is more prominent, since the quality factor is smaller towards the apex, and also the wave passes through more elements which results in more losses. Therefore, for the 40th element, the peak of the response appears to be low, as is also seen for the lower-frequency plot in Fig. 4a. The fluctuations that appear in the frequency response at the lower part of the shown spectrum are outside the working frequency range.

The phase of the pressure is plotted against the element number and against frequency in Fig. 4c, d. The phase variation up to the response peak is also similar to that in the cochlea^12^. The larger phase shift at positions or frequencies beyond the peak is less important, since the response has already decreased to very small values there. The fact that at higher frequencies the phase shift from base to peak is less than that at lower frequencies, also observed in the cochlea^12^, may be attributed to the fact that it takes a considerable number of elements for the phase to accumulate, which is prevented at high frequencies by the small number of elements involved. It is seen in the phase plots that the phase-shift parameter, $N_c$, which has the value 2.5 in this design, only approximately corresponds to the phase shift up to the peak. The obvious similarity of the coupled response with respect to the element number, *n*, and with respect to logarithmic frequency observed in both the modulus and phase plots is similar to the ‘scaling symmetry’ in the cochlea^24^. It should be noted, however, that since $Q_1$ depends both on frequency and neck radius, according to Eq. (6), and the neck radius is assumed to be constant, then $Q_1$ varies with the natural frequency of the element in this design. This means that this design does not exactly satisfy scaling symmetry, as illustrated in the plots of the response against non-dimensional quantities in Fig. S7 in the Supplementary material.

The stop bands for the three different elements are illustrated in Fig. 4b, d by shaded areas of corresponding colours. For the 40th and 26th elements, the response is seen to decay rapidly and little phase shift occurs within the stop band. Beyond the stop band, there is a second pass band, as shown in the wavenumber frequency variation in Fig. 3b, which leads to further phase shift of the response in Fig. 4d. The modulus of the response in Fig. 4b continues to decrease in the second pass band, albeit at a slower rate compared to in the stop band. For the basal element $n=12$, an inversion of the phase is observed in the stop band and slightly beyond that. This, however, corresponds to a very low response modulus and is therefore not expected to have any significant effect. Secondary peaks appear beyond about 4 kHz, outside the working frequency range, which are also significantly lower than the primary response peak.

The pressure modulus plots in Fig. 4a, b predict that the designed system can be used as an acoustic rainbow sensor, where different frequency components are filtered at different positions, since each element acts as a band-pass filter with a smooth frequency response centred at a different frequency. Therefore, the different frequency components can be captured by introducing small microphones in the cavities of the Helmholtz resonators, as was done in a system with quarter-wave resonators in^10^.

#### Variation of the number of elements

**Fig. 5:**
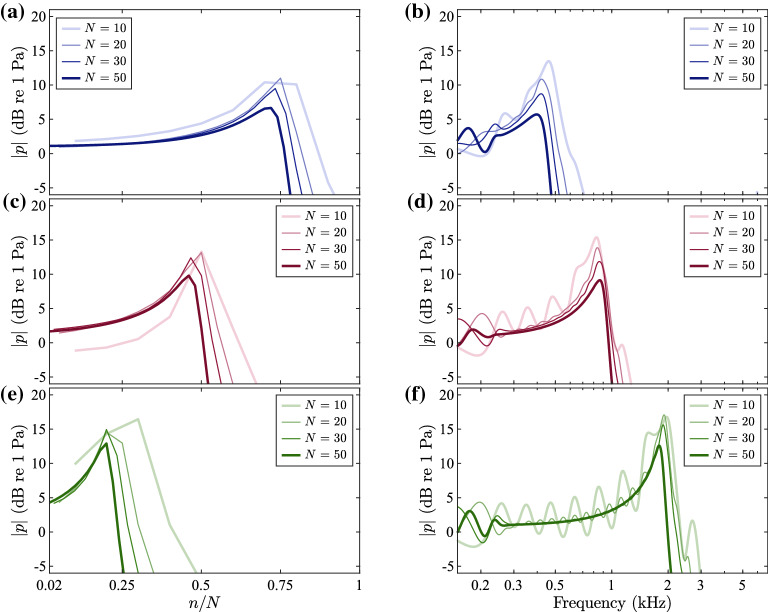
Simulations for different numbers of elements. (**a**), (**c**) and (**e**) Variation of the pressure in the resonators for different numbers of elements, *N*, at frequencies 500 Hz, 1 kHz and 2 kHz, respectively, plotted against the element number, *n*, normalised by the number of elements, *N*. (**b**), (**d**) and (**f**) Frequency response of the pressure in the resonators for different numbers of elements, *N*, at elements whose resonance frequency is the closest to 500 Hz, 1 kHz and 2 kHz, respectively; for $N=50$, these correspond to the 40th, the 26th and the 12th element.

Figure 5a, c, e show the pressure in the resonators for different values of the total number of elements, *N*, at frequencies 500 Hz, 1 kHz and 2 kHz, respectively, plotted against the element number, *n*, normalised by the total number of elements in each case. Figure 5b, d, f show the corresponding frequency response for the elements whose resonance frequencies are the closest to 500 Hz, 1 kHz and 2 kHz, respectively. For all the different values of *N*, the first and last element of the system are the same, as given in Table 1, based on the chosen working frequency band, and the intermediate elements are scaled accordingly. In Fig. 5d, the smoothness of the frequency response begins to break down when there are 30 elements, and fluctuations in the frequency response are apparent with 20 elements in all cases. It can also be observed that more elements give more suppressed peaks, due to the presence of more overall damping in the system, which is dominated by the necks of the Helmholtz resonators.

#### Absorption

**Fig. 6:**
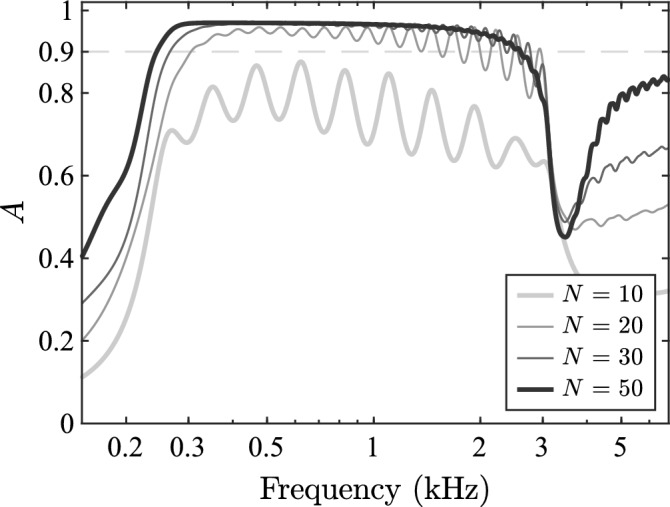
Absorption. Frequency variation of the absorption coefficient for different numbers of elements. The dashed horizontal line corresponds to a value of 0.9.

The absorption coefficient, *A*, defined as the ratio of absorbed to incident power, as presented in the Supplementary material, is plotted against frequency in Fig. 6. It can be seen that for $N=50$, incident waves are primarily absorbed within the region defined by the lowest and highest resonance frequencies, as seen from the part of the plot above the 0.9 line, which implies that the waves are neither reflected nor further transmitted. Therefore, the smooth response of the rainbow sensor, which requires that waves are concentrated at certain elements for different frequencies without reflections and without further transmission, also ensures that the system performs as an acoustic absorber with approximately uniform broadband absorption. It can be seen that using less than 50 elements produces fluctuations of the absorption coefficient with frequency, and that using less elements generally reduces the absorption.

## Discussion

A new set of design principles for an acoustic rainbow sensor has been presented, which is predicted to provide a smooth frequency response, similar to the cochlea. The system consists of a main duct with side branches of Helmholtz resonators whose dimensions vary along it, and whose main design characteristics are based on the characteristics of the cochlea. Such a system enhances and absorbs incident waves at different positions for different frequencies, and can therefore be used as an acoustic rainbow sensor, that is, as a spatial spectrum analyser. The designed rainbow sensor also behaves as a broadband acoustic absorber, with different frequency components being absorbed around their corresponding peak positions. The design procedure revealed that, for a given value of the phase-shift parameter, a compromise between the number of elements and the damping has to be made to achieve a desirable frequency selectivity. If too few elements are used, this gives rise to unevenness in the frequency response, as seen for example in^1,3,5^. Although quarter-wave resonators were used in^10^, rather than Helmholtz resonators, the equivalent criterion for a smooth frequency response is found to be satisfied for their passive system, but not for their active system. The ripples observed in the measured response are thus likely to be due to the discrete nature of their implementation, as opposed to an inherent feature in the real cochlea.

By drawing an analogy between the rainbow sensor presented above and the cochlea, the compliance of the air in the main duct of the rainbow sensor corresponds to the compliance of the fluid in the cochlear chambers, while the compliance of the resonator cavity corresponds to the compliance of the basilar membrane. In the rainbow sensor, the upper limit of the stop band is given by $f_2=f_1\sqrt{1+C_H/C_D}$. This leads to a stop band of limited bandwidth, determined by the ratio of the compliance of the air in the resonator cavity to that of the air in the main duct, which are in general of comparable size, and chosen to be equal in the design example above. In the cochlea, however, the fluid in the chambers is generally considered to be practically incompressible^12^, which corresponds to a vanishing fluid compliance, and therefore leads to an infinite bandwidth for the stop band. In this case no upper pass band would occur in the cochlea^12^.

There are around 3,000 rows of sensory, inner hair cells, approximately uniformly distributed along the human cochlea, which could be considered as the discrete elements of a Transmission Line. The audible spectrum for young people is around 10 octaves^25^, leading to about 300 elements per octave. In the passive cochlea, the local resonance quality factor is around^13^ 2.5, which, according to Eq. (8), gives a minimum requirement of only about 8 elements per octave. Even if a higher value of, for example^21^, $Q_1=50$ is used to approximate the behaviour of the active cochlea, due to the outer hair cells, with single-degree-of-freedom basilar membrane dynamics, the required number of elements per octave is found to be about 35. This is still much smaller than the 300 elements per octave present in the human cochlea. Using the nominal values $N_{oct}=300$ and $N_c=2.5$ for the cochlea in Eq. (8) also gives a maximum value for the quality factor to ensure that at least six elements per wavelength are present as $Q_1\le (N_{oct}/2N_c)^2=3600$. This suggests that extremely low values of damping would be required for reflections to occur inside the cochlea from the discontinuities of consecutive elements if their parameters are smoothly varying. In practice, however, reflections are thought to be generated by the roughness in the variation of these parameters, giving rise to certain forms of otoacoustic emission^25,26^.

## Methods

The system shown in Fig. 1a can be analysed using Transfer Matrices^7^, where each element, consisting of a duct segment and a side branch, is represented as a two-port network with a Transfer Matrix $\mathbf {T}_n$. The pressure and volume velocity at the input of an element can be expressed with respect to the corresponding quantities at its output either explicitly or compactly as^15^

10 $$\begin{aligned} \begin{bmatrix} p_n \\ q_n \end{bmatrix}= \begin{bmatrix} T_{11,n} &{} T_{12,n}\\ T_{21,n} &{} T_{22,n} \end{bmatrix} \begin{bmatrix} p_{n+1} \\ q_{n+1} \end{bmatrix} \text { and }{\mathbf {v}}_n = {\mathbf {T}}_n \mathbf {v}_{n+1}, \end{aligned}$$

where $\mathbf {v}_n$ is the state vector of the *n*-th element. The *N*-th element can be expressed with respect to the state variables at the termination as $\mathbf{v}_{N} =\mathbf{T}_N\mathbf{v}_{end}$, where $\mathbf{v}_{end}=[{p}_{end} {q}_{end}]^{\mathrm{T}}$, and ${p}_{end}$ and $q_{end}$ are the pressure and volume velocity at the termination of the system. By iteratively substituting the lower-order vector by its relation with the vector of the next element using Eq. (10), the first state vector can be expressed with respect to the last one as $\mathbf {v}_1=\mathbf {T}\mathbf {v}_{end}$, where $\mathbf {T}=\mathbf {T}_1\mathbf {T}_2\dots \mathbf {T}_{N-1}\mathbf {T}_N$ is the total Transfer Matrix and $\mathbf{v}_{1}={[{p}_{in} q_{in}]}^{\mathrm{T}}$, where $p_{in}$ and $q_{in}$ are the pressure and volume velocity at the input. Given the input volume velocity, $q_{in}$, along with the termination impedance, $Z_{end}=p_{end}/q_{end}$, the other three state variables, that is, $p_{in}$, $p_{end}$ and $q_{end}$, can be found by solving the matrix equation $\mathbf {v}_1=\mathbf {T}\mathbf {v}_{end}$ along with the expression for $Z_{end}$. The pressure and volume velocity at the input of each element can be calculated once $p_{end}$ and $q_{end}$ are found, by iteratively applying Eq. (10) starting from the last element. The pressure in the resonators can then be calculated with expressions given in the Supplementary material. Detailed formulas for the Transfer Matrices, as well as formulas for calculating the absorption coefficient, are given in the Supplementary material.

## Supplementary information

Supplementary material 1
