# Supplementary material for: Cochlea-inspired design of an acoustic rainbow sensor with a smoothly varying frequency response
Source: Sci Rep. 2020 Jul 1;10:10803. doi: 10.1038/s41598-020-67608-z (PMC7330050; doi:10.1038/s41598-020-67608-z)
Supplement: Supplementary file 1 — Supplementary material 1 [file 41598_2020_67608_MOESM1_ESM.pdf]

Supplementary material for: Cochlea-inspired design of an acoustic rainbow sensor with a smoothly varying frequency response

Angelis Karlos* and Stephen J. Elliott

Institute of Sound and Vibration Research, University of Southampton, Highfield Campus, Southampton, SO17 1BJ, United Kingdom

*Corresponding author. E-mail: A.Karlos@soton.ac.uk

S1 A lumped-parameter model of a damped Helmholtz resonator

A Helmholtz resonator is depicted in Fig. S1a, consisting of a neck of radius a and length l_a, and a cavity of volume V_H, which are all assumed to be small compared with the acoustic wavelength, so that a lumped-parameter model can be used. The main inertive element of the resonator is the inertia of the air in the neck and the main compliant element is the air in the cavity, a balance between which results in resonance [1]. The acoustic resistance in this lumped-parameter analysis is considered to be only due to viscous and thermal losses occurring at the neck boundary. An effective neck length, denoted by l_H, is used both for the inertance and the resistance of the neck, which is larger than the actual neck length, l_a, since it includes end corrections due to radiation at both sides of the neck.

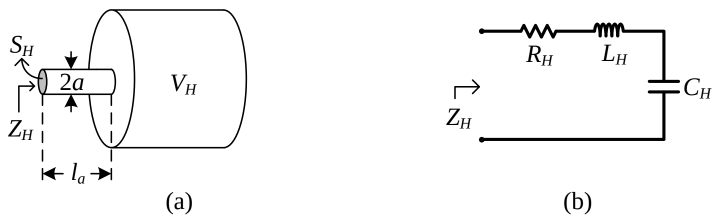

Figure S1. Helmholtz resonator. (a) Schematic of a Helmholtz resonator. The neck radius, a, length, l_a, and cross-sectional area, S_H, are shown, along with the cavity volume, V_H. (b) Equivalent circuit will lumped parameters, showing the acoustic resistance, R_H, and inertance, L_H, of the neck, and the compliance, C_H, of the cavity. The input impedance, Z_H, of the resonator is also shown in both figures.

Assuming wave propagation in the neck, the input impedance looking into the resonator, as depicted in Fig. S1a, is given by [2]

$$Z_H = Z_{0,n} \frac{Z_C + \mathrm{i} Z_{0,n} \tan k_H l_H}{Z_{0,n} + \mathrm{i} Z_C \tan k_H l_H}, \tag{S1}$$

where i is the imaginary unit and $Z_{0,n} = \rho c_0 / S_H$ is the characteristic impedance for wave propagation in the neck, where ρ is the density of air and c_0 is the speed of sound in air, k_H is a complex wavenumber accounting for both propagation and attenuation in the neck, and Z_C is the impedance of the cavity, given by

$$Z_C = \frac{1}{\mathrm{i}\omega C_H}, \tag{S2}$$

where $C_H = V_H / \rho c_0^2$ is the compliance of the cavity, where V_H is the cavity volume, and ω is the angular frequency. If the neck length is assumed to be much smaller than the wavelength, and provided that the imaginary part of the wavenumber is much smaller than its real part, it follows that $|k_H l_H| \ll 1$.

If it is also assumed that the volume of the cavity is large compared to the volume of the neck, so that $|Z_C| k_H l_H \ll Z_{0,n}$, equation (S1) takes the approximate form

$$Z_H = \mathrm{i} Z_{0,n} k_H l_H + Z_C. \tag{S3}$$

The viscous boundary layer thickness for sound propagation in the neck is given by [3]

$$\delta = \sqrt{\frac{2\eta}{\omega\rho}}, \tag{S4}$$

where η is the dynamic viscosity. Assuming that the neck radius is much greater than the viscous boundary layer thickness, $a \gg \delta$, the wavenumber for sound propagation in the neck can be written as [3]

$$k_H = k_0 - \mathrm{i}\alpha, \tag{S5}$$

where $k_0 = \omega/c_0$ is the lossless wavenumber and α is the loss coefficient, which represents the spatial exponential decay rate, given by [3]

$$\alpha = \frac{k_0 \delta}{2a}\left(1 + \frac{\gamma - 1}{\sqrt{\mathrm{Pr}}}\right), \tag{S6}$$

where γ is the ratio of specific heats and Pr is the Prandtl number. In equation (S6), the first term in parentheses multiplied by the common term corresponds to viscous losses, and the second term multiplied by the common term corresponds to thermal losses. For typical parameter values for air at room temperature ($\rho = 1.2$ kg·m^{-3}, $c_0 = 343$ m·s^{-1}, $\eta = 1.8 \cdot 10^{-5}$ Pa·s, $\gamma = 1.4$, Pr $= 0.71$ [4]), the loss coefficient can be written approximately as

$$\alpha \approx 3 \cdot 10^{-5} \frac{\sqrt{f/\mathrm{Hz}}}{a}, \tag{S7}$$

where f is in Hz, so that if a is in meters then α is in Np/m.

Substituting equation (S5) into equation (S3), the impedance of the Helmholtz resonator takes the form

$$Z_H = \mathrm{i}\frac{\rho c_0 l_H}{S_H}(k_0 - \mathrm{i}\alpha) + Z_C = R_H + \mathrm{i}\omega L_H + \frac{1}{\mathrm{i}\omega C_H}, \tag{S8}$$

where

$$R_H = \frac{\alpha \rho c_0 l_H}{S_H} = Z_{0,n}\alpha l_H,\ L_H = \frac{\rho l_H}{S_H},\ C_H = \frac{V_H}{\rho c_0^2} \tag{S9}$$

are the acoustic resistance and inertance of the neck, and the compliance of the cavity, respectively. Equation (S8) is equivalent to a lumped-parameter representation of the Helmholtz resonator, an equivalent circuit of which is depicted in Fig. S1b.

S2 End corrections for the neck of a Helmholtz resonator

The effective length of the neck is equal to the sum of its physical length and end corrections at both ends. For the inertance, these end corrections account for the mass of air outside the neck that gets accelerated by the air within it [3], whereas for the resistance, the end corrections account for the additional loss as the air flows around the two ends of the neck [1]. The well-known results for the end correction are that

it is approximately $0.6a$ for an unflanged end and $0.85a$ for a flanged end. In the design considered here, the baffle is large relative to the size of the neck, so that the total end correction length, accounting for both ends of the neck, can be approximately calculated by [1]

$$l_{cor} \approx 2 \cdot 0.85a = 1.7a. \tag{S10}$$

The effective neck length is thus related to the actual neck length by $l_H = l_a + l_{cor}$. More complicated formulas have also been proposed for a baffle of finite size, so that the end corrections from the neck to the cavity and from the neck to the main duct can be calculated, respectively, by [5]

$$l_{cor,c} = 0.82 \left[1 - 1.35\frac{a}{r_c} + 0.31\left(\frac{a}{r_c}\right)^3\right] a \tag{S11}$$

and

$$l_{cor,d} = 0.82 \left[1 - 0.235\frac{a}{r_d} - 1.32\left(\frac{a}{r_d}\right)^2 + 1.54\left(\frac{a}{r_d}\right)^3 - 0.86\left(\frac{a}{r_d}\right)^4\right] a, \tag{S12}$$

where r_c and r_d are the hydraulic radii of the cavity and the duct, respectively, and a is the neck radius. The total end correction length is then given by $l_{cor} = l_{cor,c} + l_{cor,d}$. The hydraulic radius is defined by [1]

$$r_j = 2\frac{S_{cs,j}}{P_{cs,j}}, \tag{S13}$$

where $S_{cs,j}$ and $P_{cs,j}$ are the area and the perimeter of the cross section, and the subscript j can vary to denote either the cavity or the duct in this case. The hydraulic radius of a duct of circular cross section coincides with its geometric radius, and that of a duct with square cross section of side w is equal to $w/2$.

S3 Transfer Matrices and calculation of the pressure in the cavities of the Helmholtz resonators

A schematic of a system consisting of a main duct with N side branches of Helmholtz resonators of varying dimensions, driven by a semi-infinite uniform duct of characteristic impedance Z_0 and terminated at an impedance Z_{end}, is shown in Fig. S2a. One element of the system consists of a duct segment and a Helmholtz resonator. The system of Fig. S2a can be analysed using Transfer Matrices [4]. An equivalent drawing of the system is shown in Fig. S2b, where each element is represented as a two-port network, where the Transfer Matrix of the n-th element is $\mathbf{T}_n$. The pressure and volume velocity at the input of an element can be expressed with respect to the corresponding quantities at its output either explicitly or compactly as [6]

$$\begin{bmatrix} p_n \\ q_n \end{bmatrix} = \begin{bmatrix} T_{11,n} & T_{12,n} \\ T_{21,n} & T_{22,n} \end{bmatrix} \begin{bmatrix} p_{n+1} \\ q_{n+1} \end{bmatrix}, \text{ and } \mathbf{v}_n = \mathbf{T}_n \mathbf{v}_{n+1}, \tag{S14}$$

respectively, where $\mathbf{v}_n$ is the state vector of the n-th element. The N-th state vector can be expressed with respect to the state vector at the termination as $\mathbf{v}_N = \mathbf{T}_N \mathbf{v}_{end}$, where $\mathbf{v}_{end} = [p_{end}\ q_{end}]^{\mathrm{T}}$. By iteratively substituting the lower-order vector by its relation with the vector of the next element using equation (S14), the first state vector can be expressed with respect to the last one as $\mathbf{v}_1 = \mathbf{T}\mathbf{v}_{end}$, where

$\mathbf{T} = \mathbf{T}_1\mathbf{T}_2 \dots \mathbf{T}_{N-1}\mathbf{T}_N$ is the total Transfer Matrix, or, in expanded form as

$$\begin{bmatrix} p_{in} \\ q_{in} \end{bmatrix} = \begin{bmatrix} T_{11} & T_{12} \\ T_{21} & T_{22} \end{bmatrix} \begin{bmatrix} p_{end} \\ q_{end} \end{bmatrix}. \tag{S15}$$

Given the input volume velocity, q_{in}, along with the termination impedance, $Z_{end} = p_{end}/q_{end}$, the other three state variables, that is, p_{in}, p_{end} and q_{end}, can be found by solving the system of equations (S15) along with the expression for Z_{end}. The input impedance of the system can then be calculated by $Z_{in} = p_{in}/q_{in}$. The pressure and volume velocity at the input of each element can be calculated once p_{end} and q_{end} are found, by iteratively applying equation (S14) starting from the last element.

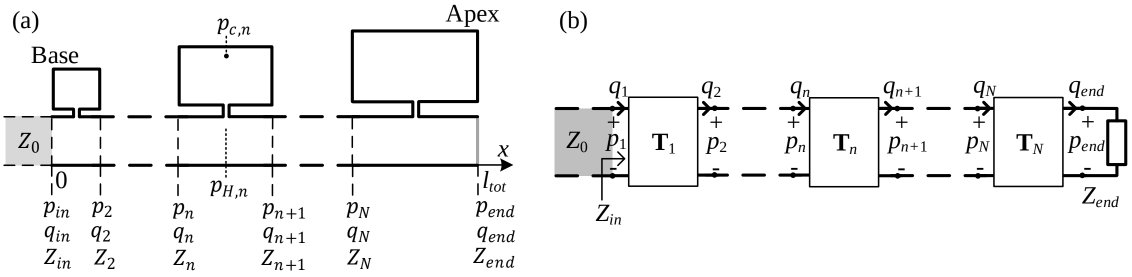

Figure S2. Schematic of an acoustic rainbow sensor. Longitudinal cross section of a system consisting of a main duct with N side branches of Helmholtz resonators of varying dimensions. Each element of the system consists of a duct segment and a Helmholtz resonator. The system is connected to a semi-infinite waveguide of characteristic impedance Z_0, and terminates at an impedance Z_{end}. The pressure in the main duct at the position of the n-th side branch, $p_{H,n}$, and the pressure at the top of the n-th cavity, $p_{c,n}$, are shown. The base and the apex correspond to the input and output ends, respectively. (b) Representation of the system where each element is represented by a two-port network. The two-port network of the n-th element has a Transfer Matrix $\mathbf{T}_n$, input variables p_n and q_n and output variables p_{n+1} and q_{n+1}.

The Transfer Matrix relating the state variables at the two sides of the n-th element can be expressed as a product of Transfer Matrices, accounting for wave propagation through the n-th duct segment and for the effect of the Helmholtz resonator side branch, as $\mathbf{T}_n = \mathbf{T}_{hd,n}\mathbf{T}_{H,n}\mathbf{T}_{hd,n}$ [6], where $\mathbf{T}_{hd,n}$ is the Transfer Matrix of half the duct segment of the element and $\mathbf{T}_{H,n}$ is the Transfer Matrix for the Helmholtz resonator. The Transfer Matrix for half the duct segment of the n-th element and the Transfer Matrix of the side branch are written as [6]

$$\mathbf{T}_{hd,n} = \begin{bmatrix} \cos\frac{k_0 l_{D,n}}{2} & \mathrm{i}Z_0 \sin\frac{k_0 l_{D,n}}{2} \\ \frac{\mathrm{i}}{Z_0}\sin\frac{k_0 l_{D,n}}{2} & \cos\frac{k_0 l_{D,n}}{2} \end{bmatrix} \text{ and } \mathbf{T}_{H,n} = \begin{bmatrix} 1 & 0 \\ \frac{1}{Z_{H,n}} & 1 \end{bmatrix}, \tag{S16}$$

respectively, where $Z_{H,n}$ is the impedance of the n-th Helmholtz resonator. Transfer Matrices can also be used for the calculation of the impedance of the Helmholtz resonator, $Z_{H,n}$. The Transfer Matrices for the neck, the end corrections and the cavity are given, respectively, by [7]

$$\mathbf{T}_{neck,n} = \begin{bmatrix} \cos k_H l_{a,n} & \mathrm{i}Z_{neck,0} \sin k_H l_{a,n} \\ \frac{\mathrm{i}\sin k_H l_{a,n}}{Z_{neck,0}} & \cos k_H l_{a,n} \end{bmatrix}, \tag{S17}$$

$$\mathbf{T}_{cor,n} = \begin{bmatrix} 1 & \mathrm{i}Z_{neck,0} \sin k_H l_{cor} \\ 0 & 1 \end{bmatrix} \tag{S18}$$

and

$$\mathbf{T}_{cav,n} = \begin{bmatrix} \cos k_{c,n}h_H & \mathrm{i}Z_{c,0,n}\sin k_{c,n}h_H \\ \frac{\mathrm{i}\sin k_{c,n}h_H}{Z_{c,0,n}} & \cos k_{c,n}h_H \end{bmatrix}, \tag{S19}$$

where k_H is the wavenumber of the necks, $l_{a,n}$ is the actual length of the neck of the n-th element, $k_{c,n}$ and $h_{H,n}$ are the wavenumber and the height, respectively, of the cavity of the n-th element, l_{cor} is the total end correction length, $Z_{neck,0}$ is the characteristic impedance of the necks and $Z_{c,0,n}$ is the characteristic impedance of the n-th cavity. The total end correction, l_{cor}, is calculated as the sum of the two end corrections given in equations (S11) and (S12). The wavenumber and the characteristic impedance of the neck are the same for all elements due to the necks having the same cross section.

The state vector at the input of the Helmholtz resonator is related to the state vector at the top of the cavity of the resonator by [7]

$$\mathbf{v}_{H,n} = \mathbf{T}_{neck,n}\mathbf{T}_{cor,n}\mathbf{T}_{cav,n}\mathbf{v}_{c,n}, \tag{S20}$$

where $\mathbf{v}_{H,n} = [p_{H,n}\ q_{H,n}]^{\mathrm{T}}$ and $\mathbf{v}_{c,n} = [p_{c,n}\ q_{c,n}]^{\mathrm{T}}$ are the state vectors at the input and at the top of the cavity of the Helmholtz resonator, respectively. Considering that the volume velocity at the top of the cavity, $q_{c,n}$, is zero, and that the impedance of the Helmholtz resonator is given by $Z_{H,n} = p_{H,n}/q_{H,n}$, yields [7]

$$Z_{H,n} = -\mathrm{i}\frac{\cos k_H l_{a,n}\cos k_{c,n}h_{H,n} - \frac{Z_{neck,0}}{Z_{c,0,n}}\sin k_{c,n}h_{H,n}\left(k_H l_{cor}\cos k_H l_{a,n} + \sin k_H l_{a,n}\right)}{\frac{1}{Z_{neck,0}}\sin k_H l_{a,n}\cos k_{c,n}h_{H,n} - \frac{1}{Z_{c,0,n}}\sin k_{c,n}h_{H,n}\left(k_H l_{cor}\sin k_H l_{a,n} - \cos k_H l_{a,n}\right)}. \tag{S21}$$

The pressure in the main duct at the position of the side branches, that is, half way through the element length, can be calculated by

$$p_{H,n} = \mathbf{T}_{hd,n}(1,1)p_{n+1} + \mathbf{T}_{hd,n}(1,2)q_{n+1}, \tag{S22}$$

where $\mathbf{T}_{hd,n}(1,1)$ and $\mathbf{T}_{hd,n}(1,2)$ denote the element on the first row and first column and the element on the first row and second column, respectively. The pressure at the top of the cavity can be calculated from equation (S20) with $q_{c,n} = 0$, giving

$$p_{c,n} = \frac{p_{H,n}}{\cos k_H l_{a,n}\cos k_{c,n}h_{H,n} - \frac{Z_{neck,0}}{Z_{c,0,n}}\sin k_{c,n}h_{H,n}\left(k_H l_{cor}\cos k_H l_{a,n} + \sin k_H l_{a,n}\right)}. \tag{S23}$$

The losses can be calculated by using complex wavenumbers and characteristic impedances in the Transfer Matrices above, which are given, with minor corrections, by [5]

$$k_j = k_0\left[1 + (1-\mathrm{i})\frac{\delta}{2r_j}\left(1 + \frac{\gamma-1}{\sqrt{\mathrm{Pr}}}\right)\right] \text{ and } Z_j = Z_0\left[1 + (1-\mathrm{i})\frac{\delta}{2r_j}\left(1 - \frac{\gamma-1}{\sqrt{\mathrm{Pr}}}\right)\right], \tag{S24}$$

where δ is the viscous boundary layer thickness, given by equation (S4), and r_j is the hydraulic radius, given by equation (S13), where now the subscript j can take different values to denote the neck, the cavity or the main duct.

S4 Calculation of the absorption coefficient

The reflection coefficient at the input of the system can be calculated by $R = p_{in}^-/p_{in}^+$, where p_{in}^+ is the incident pressure wave at the input boundary, $x = 0$, and p_{in}^- is the reflected wave at $x = 0$. The total pressure and volume velocity at $x = 0$ are given, respectively, by $p_{in} = p_{in}^+ + p_{in}^-$ and $q_{in} = \left(p_{in}^+ - p_{in}^-\right)/Z_0$.

Using these relations along with equation (S15) and the definition of the reflection coefficient above, the latter can be written as

$$R = \frac{T_{11} + \frac{T_{12}}{Z_{end}} - Z_0 T_{21} - \frac{Z_0}{Z_{end}} T_{22}}{T_{11} + \frac{T_{12}}{Z_{end}} + Z_0 T_{21} + \frac{Z_0}{Z_{end}} T_{22}}. \tag{S25}$$

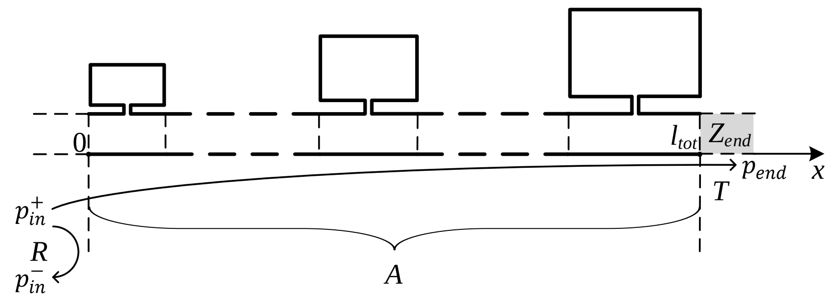

Figure S3. Schematic of a waveguide with an array of varying Helmholtz resonators. The waveguide is terminated by a waveguide of characteristic impedance Z_{end}. The incident, p_{in}^+, and reflected, p_{in}^-, pressure components at the input are shown, are shown, as well as the reflection, R, transmission, T, and absorption, A, coefficients.

The transmission coefficient is defined as the ratio of the transmitted wave beyond the end of the waveguide to the incident wave at the input, $T = p_{end}/p_{in}^+$, where p_{end} is the transmitted pressure at the output. The volume velocity at $x = l_{tot}$ is given by $q_{end} = p_{end}/Z_{end}$. Combining these relations with equation (S15) and with the definition of the transmission coefficient above yields

$$T = \frac{2}{T_{11} + \frac{T_{12}}{Z_{end}} + Z_0 T_{21} + \frac{Z_0}{Z_{end}} T_{22}}. \tag{S26}$$

The absorption coefficient is the ratio of the power absorbed within the waveguide to the incident power, so that it can be calculated by $A = 1 - R_p - T_p$, where R_p and T_p are the power reflection coefficient and the power transmission coefficient, defined as the ratios of the reflected and the transmitted power, respectively, to the incident power, given by $R_p = |R|^2$ and $T_p = |T|^2 Z_0/Z_{end}$ [1]. The absorption coefficient can thus be written with respect to the amplitude reflection and transmission coefficients, as

$$A = 1 - |R|^2 - \frac{Z_0}{Z_{end}} |T|^2. \tag{S27}$$

For a waveguide terminated at an impedance $Z_{end} = Z_0$, which matches the characteristic impedance of the main duct, the absorption coefficient becomes $A = 1 - |R|^2 - |T|^2$ and the reflection and transmission coefficients of equations (S25) and (S26) are written as

$$R = \frac{T_{11} + \frac{T_{12}}{Z_0} - Z_0 T_{21} - T_{22}}{T_{11} + \frac{T_{12}}{Z_0} + Z_0 T_{21} + T_{22}} \text{ and } T = \frac{2}{T_{11} + \frac{T_{12}}{Z_0} + Z_0 T_{21} + T_{22}}. \tag{S28}$$

For a rigid termination, where $Z_{end} \to \infty$, the power transmission coefficient vanishes, so that the absorption coefficient of equation (S27) takes the form $A = 1 - |R|^2$, and the reflection coefficient of equation (S25) becomes $R = (-Z_0 + T_{11}/T_{21})/(Z_0 + T_{11}/T_{21})$.

S5 Simulations of the wavenumber and the propagation velocities

An equivalent wavenumber, k, can be calculated for the system, if it is assumed that the forward-travelling pressure and volume velocity change from the input end of the n-th element to its output by a factor of $\psi_n = \exp(-ik_n\Delta x_n)$, and the respective backward-travelling components by a factor of $\psi_n^{-1} = \exp(ik_n\Delta x_n)$, where $\Delta x_n = x_{n+1} - x_n$ for the n-th element. This assumption is equivalent to the factors ψ_n and ψ_n^{-1} being the eigenvalues of the n-th Transfer Matrix given in equation (S14), as explained in [8]. The wavenumber for the forward-travelling wave at the n-th element can be calculated as

$$k_n = \mathrm{i}\frac{\ln \psi_n}{\Delta x_n}, \tag{S29}$$

where for each element, the appropriate eigenvalue is used, so that the real part of the wavenumber is positive and the imaginary part is negative, to account for forward propagation and forward attenuation of the wave in the duct, respectively. The phase and group velocities at the n-th element can be calculated from the wavenumber by $c_{ph,n} = \omega/\mathrm{Re}\{k_n\}$ and $c_{gr,n} = \mathrm{d}\omega/\mathrm{d}(\mathrm{Re}\{k_n\})$.

The real and imaginary parts of the wavenumber and the phase and group velocities are plotted in Fig. S4, both against the element number, n, and against frequency. The solid lines in the plots are calculated from equation (S29), where equations (S24) have been used for the wavenumbers and characteristic impedances of the duct segments, resonator necks and resonator cavities in the Transfer Matrices. The dotted lines in the wavenumber plots are calculated with equation (4) in the main article, also shown in Fig. 3, which is based on a lumped-parameter analysis, using the simpler model of equations (S5) and (S6) for the wavenumber in the neck given in Section S1 above. This approach neglects the variation of the wave speed with the frequency-dependent boundary layer thickness, and also neglects the losses in the main duct and in the cavities.

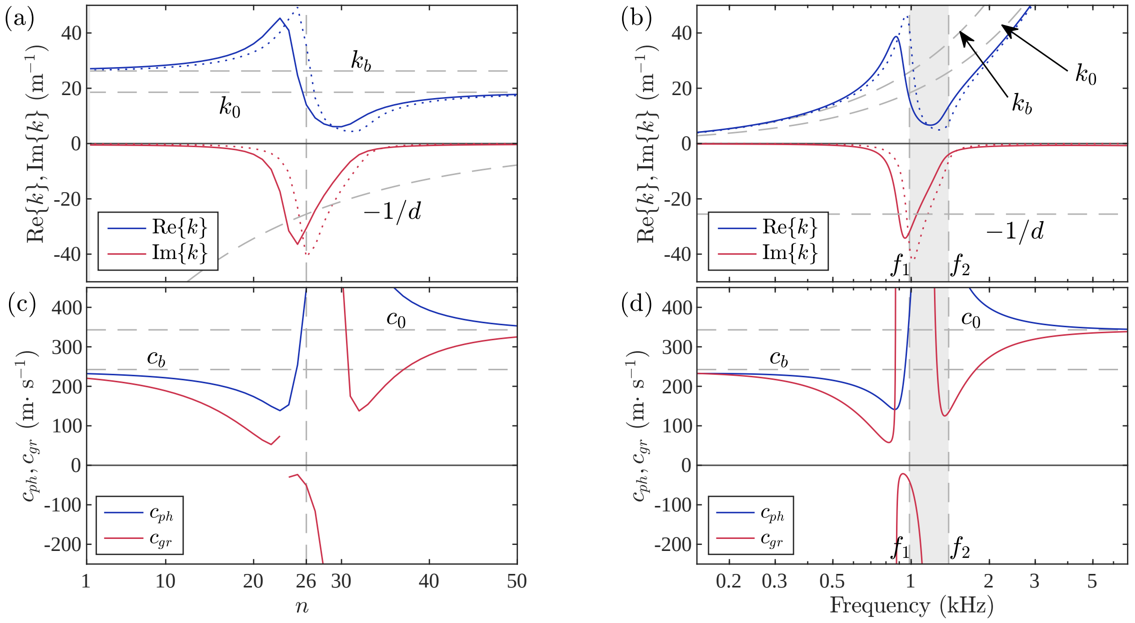

Figure S4. Wavenumber and propagation velocities. (a) Real and imaginary parts of the wavenumber at a frequency of 1 kHz, plotted against the element number, n. (b) Real and imaginary parts of the waveunmber for the 26th element, plotted against frequency. (c) Phase and group velocities at a frequency of 1 kHz, plotted against the element number, n. (d) Phase and group velocities for the 26th element, plotted against frequency. The wavenumber is calculated with equation (S29), and the phase and group velocities are then calculated from $c_{ph,n} = \omega/\mathrm{Re}\{k_n\}$ and $c_{gr,n} = \mathrm{d}\omega/\mathrm{d}(\mathrm{Re}\{k_n\})$. The dotted lines in (a) and (b) are calculated with the Transmission Line wavenumber of equation (4) in the main article, and thus coincide with those in Figs. 3a and 3b. The stop band, defined by the frequencies f_1 and f_2, is shaded in grey in (b) and (d).

The variation of the phase and group velocities against the element number, n, for a given frequency, plotted in Fig. S4c implies that the wave slows down before the element of resonance, beyond which it is greatly attenuated, as indicated by the imaginary part of the wavenumber there. The very large phase velocity and negative group velocity, indicative of the wave nearly halting [1] and of strong 'pulse reshaping' [9], respectively, are observed both in the spatial and frequency plots in Figs. S4c and S4d, so that, apart from the stop band observed in frequency, a corresponding stop region in space also appears.

S6 Results for a rigid termination

Results for the modulus and phase of the pressure in the resonators are shown in Fig. S5, both for a matched termination, that is, a semi-infinite continuation of the main duct, and for a rigid termination. The spatial response at a frequency of 300 Hz, which corresponds to the natural frequency of the last element, and the frequency response of the last element are also shown. It can be seen that the rigid termination in practice only affects the frequency response of the last element, which is anyway at the low end of the working frequency range, where the band-pass behaviour of the response is compromised by the fluctuations below. Some fluctuations also appear in the modulus and phase frequency response beyond the peak, where the modulus is already greatly decreased. The absorption coefficient is plotted in Fig. S6, where it is seen that within the working frequency range, the rigid termination does not affect the absorption.

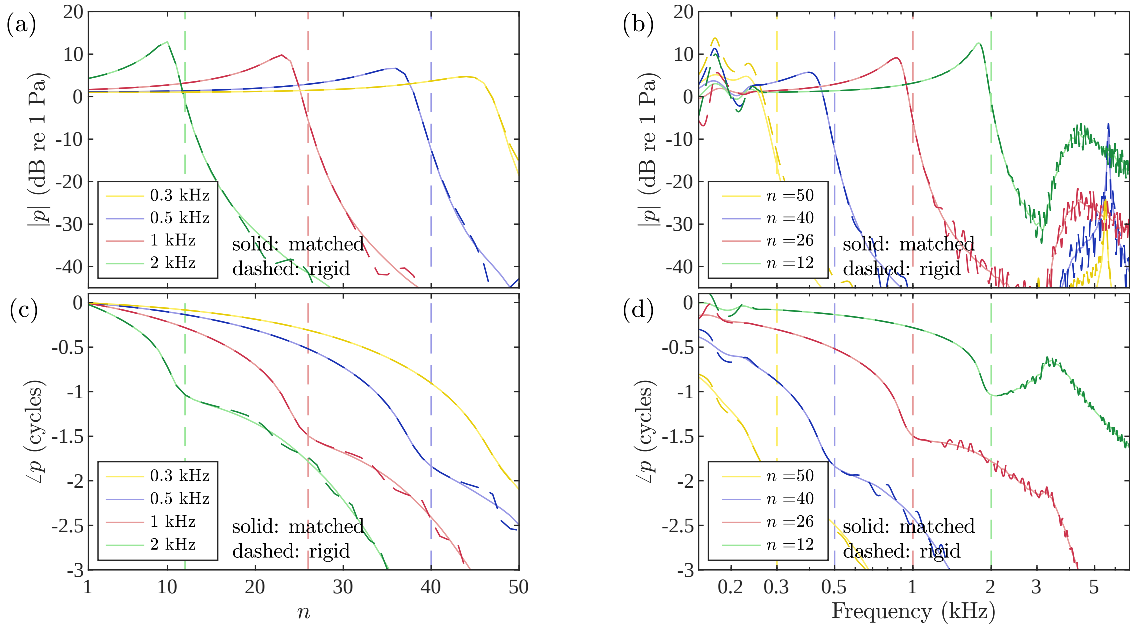

Figure S5. Pressure response with a rigid termination. (a) Modulus of the pressure in the resonators plotted against the number of element, n, for four different frequencies. (b) Modulus of the pressure in the resonators plotted against frequency for four different elements. (c) Phase of the pressure in the resonators plotted against n for four different frequencies. (d) Phase of the pressure in the resonators plotted against frequency for four different elements. In (a) and (c), the vertical lines correspond to the elements whose resonance frequency is the closest to the respective input frequency; specifically, elements 12, 26, 40 and 50 have resonance frequencies 1.97 kHz, 985 Hz, 492 Hz and 300 Hz, respectively. In (b) and (d), the vertical lines correspond to the resonance frequencies of the elements used. In all cases, the pressure with a matched termination is also plotted for reference.

S7 'Scaling symmetry' of the response

The frequency response and the spatial response of the coupled cochlea are related by a 'scaling symmetry'. By virtue of this symmetry, the coupled response at a specific position plotted against the frequency

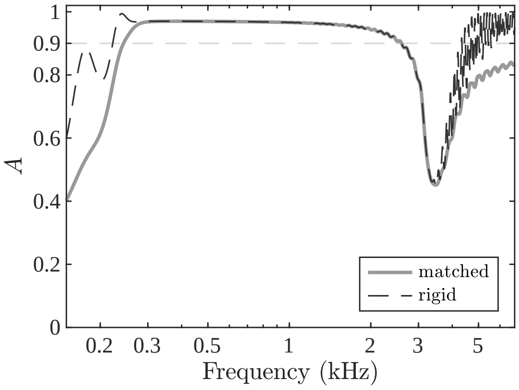

Figure S6. Absorption. Frequency variation of the absorption coefficient both for a matched and for a rigid termination.

normalised by the characteristic frequency of that position is similar to the coupled response at a specific frequency normalised by the spatially varying local characteristic frequency, where the characteristic frequency of a given position is the frequency of peak response [10]. The same principle applies to the designed rainbow sensor within its working range. This can be seen in the similarity between the modulus and phase plots of Fig. S7 against non-dimensional quantities, where the response for three different excitation frequencies is plotted against the excitation frequencies normalised by the resonator-dependent characteristic frequency, $f_i/\mathrm{CF(n)}$, in Figs. S7a and S7c, and the response at three different resonators is plotted against the frequency normalised by the characteristic frequencies of the specific resonators, $f/\mathrm{CF(n_i)}$, in Figs. S7b and S7d. The symmetry is more accurate when the specific position and frequency are linked by the tonotopic mapping, as can be seen for the same-colour plots in the left and right graphs of the figure. The difference in the response when the excitation frequency is far from the resonance frequency of the specific element is due to the variation of the resonance quality factor between the resonators, according to equation (6) in the main article.

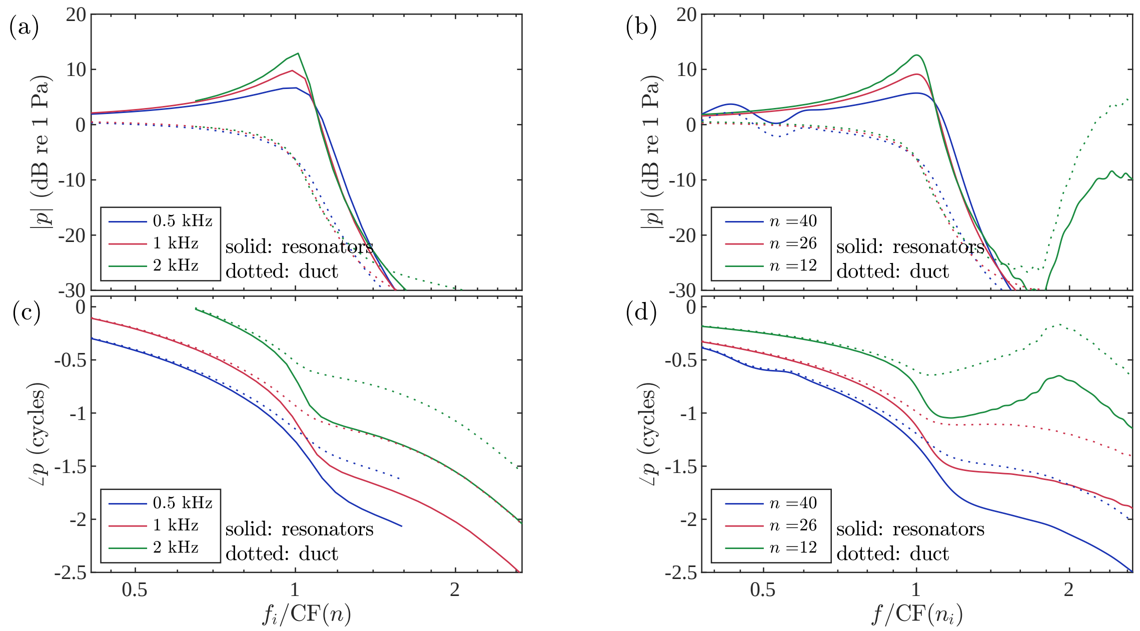

Figure S7. Pressure response against non-dimensional quantities. (a) Modulus and (c) phase of the coupled response for three different excitation frequencies plotted against the excitation frequencies normalised by the resonator-dependent characteristic frequency. (b) Modulus and (d) phase of the coupled response at three different resonators plotted against the frequency normalised by the characteristic frequencies of the specific resonators. Elements 12, 26 and 40 have resonance frequencies 1.97 kHz, 985 Hz and 492 Hz, respectively.

References

[1] D. T. Blackstock. *Fundamentals of Physical Acoustics.* Wiley and Sons Ltd, New York, 2000.

[2] H. F. Olson. *Acoustical Engineering.* D. Van Nostrand Company, Inc., USA, 1957.

[3] L. E. Kinsler, A. R. Frey, A. B. Coppens, and J. V. Sanders. *Fundamentals of Acoustics, 4th Edition.* Wiley-VCH, United States of America, 1999.

[4] N. Jimenez, V. Romero-Garcia, V. Pagneux, and J. P. Groby. Rainbow-trapping absorbers: Broadband, perfect and asymmetric sound absorption by subwavelength panels for transmission problems. *Sci Rep*, 7(1):13595, 2017.

[5] G. Theocharis, O. Richoux, V. R. García, A. Merkel, and V. Tournat. Limits of slow sound propagation and transparency in lossy, locally resonant periodic structures. *New Journal of Physics*, 16(9), 2014.

[6] M. L. Munjal. *Acoustics of ducts and mufflers with application to exhaust and ventilation system design.* John Wiley & Sons, Inc, 1987.

[7] O. Richoux and V. Pagneux. Acoustic characterization of the Hofstadter butterfly with resonant scatterers. *Europhysics Letters*, 59(1):34–40, 2002.

[8] S. J. Elliott, G. Ni, B. R. Mace, and B. Lineton. A wave finite element analysis of the passive cochlea. *J Acoust Soc Am*, 133(3):1535–45, 2013.

[9] C. Croënne, E. J. S. Lee, Hefei Hu, and J. H. Page. Band gaps in phononic crystals: Generation mechanisms and interaction effects. *AIP Advances*, 1(4), 2011.

[10] C. A. Shera. Laser amplification with a twist: traveling-wave propagation and gain functions from throughout the cochlea. *J Acoust Soc Am*, 122(5):2738–58, 2007.
